# Thigh Abscess Caused by *Yersinia enterocolitica* in an Immunocompetent Host

**DOI:** 10.1155/2012/259475

**Published:** 2012-03-26

**Authors:** Purva Gumaste, V. Subbarao Boppana, Amarinder Singh Garcha, Donald Blair

**Affiliations:** Department of Internal Medicine, State University of New York Upstate Medical University, Syracuse, NY 13210-2342, USA

## Abstract

*Yersinia enterocolitica* is primarily a gastrointestinal tract pathogen known to cause gastroenteritis, although it may produce extra-intestinal infections like sepsis and its sequelae. However, primary cutaneous infections are extremely rare. We present a case of *Y. enterocolitica* thigh abscess in an immunocompetent adult. The portal of entry is unclear in this case. He did many outdoor activities that involved skin injuries and exposure to soil and contaminated water. Hence, direct inoculation as a result of exposure to contaminated water is postulated in the absence of evidence for a gastrointestinal route of infection.

## 1. Introduction

*Yersinia enterocolitica* is primarily a gastrointestinal tract pathogen known to cause gastroenteritis, although it may produce extraintestinal infections like sepsis and its sequelae. However, cutaneous infections are extremely rare. We present a case of *Yersinia enterocolitica* cutaneous abscess in an immunocompetent adult.

## 2. Case Report

A 54-year-old healthy man presented to the hospital with swelling and erythema of the medial aspect of his right thigh of one-week duration. The swelling was progressively worsening with increased tenderness, fever of 101 degrees F, chills, and rigors. He denied other systemic or gastrointestinal symptoms. Physical exam disclosed a well-circumscribed subcutaneous swelling measuring 4 × 3 cm on the medial aspect of right thigh below the inguinal ligament. It was mildly tender and erythematous; right inguinal lymphadenopathy was found.

The peripheral white cell blood count was slightly elevated at 10.6 with no left shift. The C-reactive protein and erythrocyte sedimentation rate were elevated at 70.8 and 53, respectively. Blood cultures were negative. Computerized tomography scan showed two small subcutaneous abscesses in the upper thigh. Aspiration yielded purulent material with a moderate number of white blood cells and no bacteria on Gram stain. The culture grew* Yersinia enterocolitica *that was confirmed and serotyped by the NY State Department of Health as O:8 using a slide agglutination test. The organism was sensitive to trimethoprim-sulfamethoxazole, ciprofloxacin, ceftazidime, ceftriaxone, imipenem, gentamicin, and tobramycin and resistant to amoxicillin/sulbactam, cefazolin, and cefoxitin.

The patient denied recent travel or consumption of pork chitterlings. He remained febrile on intravenous cefazolin, but responded quickly to ceftriaxone with complete recovery with two weeks therapy. Iron studies and hepatitis panel did not reveal any abnormalities. Stool cultures and investigations for immune compromise were not done based on the lack of history to suggest risk factors.

## 3. Discussion

*Yersinia enterocolitica,* a member of Enterobacteriaceae family, is a non-lactose fermenting Gram-negative bacillus. The most common serotypes in the United States, O:8, O:5,27, and O:1,2,3 [1], cause sporadic illness and occasional food-borne outbreaks. Serogroup O:8 may produce enteritis or mesenteric lymphadenitis in humans [2]. This strain has mainly been traced to pigs. Outbreaks have involved milk products, tofu, untreated spring water, and bean sprouts [2]. The portal of entry for this organism is the gastrointestinal tract.

Metastatic infections following septicemia with development of hepatic or splenic abscesses, pneumonia, meningitis, or osteomyelitis especially in immunocompromised host or in those with iron overload have been described [2]. Primary soft tissue infection by *Yersinia enterocolitica*, including abscess formation, without evidence of gastrointestinal or septicemia are extremely rare. While primary soft tissue infection has been documented in patients with underlying chronic illness like diabetes mellitus and hepatitis C [1–5], to our knowledge only two other cases in immunocompetent hosts, axillary and facial abscesses, have been published in English-language literature [3].

The portal of entry is unclear in this case. The patient used well water for drinking purposes, but the infecting organism was not isolated from the well water. He did many outdoor activities including mountain biking and hiking that involved many abrasions and skin injuries and exposure to soil and contaminated water. Hence, direct inoculation of *Yersinia enterocolitica* as a result of exposure to contaminated water is postulated in the absence of evidence for a gastrointestinal route of infection.
